# Effects of different patterns of movement for correcting a deep curve of Spee with clear aligners on the anterior teeth: a finite element analysis

**DOI:** 10.1186/s12903-024-03906-6

**Published:** 2024-02-10

**Authors:** Lin Zhu, Lin Liu, Wei Wang, Wen Wen Deng

**Affiliations:** 1 Graduate School of Dalian Medical University, Dalian, China; 2 Department of Orthodontics, DaLian Stomatological Hospital, DaLian, China; 3 Urumql DW Innovation InfoTech Co., Ltd., Urumqi, Xinjiang China

**Keywords:** Clear aligners, Deep curve of Spee, Different pattern of mandibular tooth movement, Effects of anterior teeth, FEM analysis

## Abstract

**Objective:**

To analyse the anterior teeth effects of clear aligners on five different patterns of mandibular molar movement and to define the most effective configuration to be implemented with clear aligners through finite element analysis.

**Methods:**

A three-dimensional mandibular model with a deep overbite in the mandible was constructed using cone beam computerized tomography (CBCT) data. The model included the mandibular dentition, mandibular periodontal ligaments, attachments, and aligners. Five models were created: (1) configuration A: second molar distalization (0.25 mm); (2) configuration B: second molar distalization (0.25 mm), first molar extrusion (0.15 mm); (3) configuration C: second molar distalization (0.25 mmm), first and second premolar extrusion(0.15 mm); (4) configuration D: second molar distalization (0.25 mm), first molar and first/second premolar extrusion(0.15 mm); and (5) configuration E: second molar distalization (0.25 mm), first molar and first/second premolar extrusion (0.15 mm), first molar and first/second premolar expansion (0.15 mm).

**Results:**

In all configurations, the anterior teeth exhibited labial tipping and the mandibular central incisor of configuration E showed the highest labial tipping. Configuration E demonstrated a relatively minor impact on mandibular molars distalization compared with configuration A. Configuration A showed the highest distal displacement value, and configuration E produced the lowest displacement value. Configuration E caused the highest periodontal ligament (PDL) pressure of the central and lateral incisors. The differences in the canines between configurations C and D,were not significant, and the stress distribution differed among the five groups.

**Conclusions:**

All patterns utilizing clear aligners facilitated mandibular molar distalization. Extruding the premolars and second molar distalization at the same time had little impact on second molar distalization; When expansion and extrusion were simultaneously performed during the distalization of mandibular molars, our prime consideration was the alveolar bone on the labial side of the anterior teeth to prevent the occurrence of gingival recession, dehiscence, and fenestration. Due to the lack of consideration for periodontal tissues in this study, clinical protocols should be designed based on the periodontal status of the mandibular anterior teeth.

## Introduction

The demand for clear aligners has increased significantly over the last decade, primarily due to their aesthetic appeal, comfort, and superior facilitation of oral hygiene compared to traditional appliances [1–3]. Clear aligners offer a desirable alternative to traditional braces, particularly for adults who prioritize the aesthetic aspects of orthodontic treatment [4, 5].

Clear aligners have been found to be highly efficient in various orthodontic treatments, including molar distalization, space closure, and anterior tooth alignment [6–8]. Previous studies have reported that the distalization movement of upper molars with clear aligners has a high predictability (approximately 80%) [8, 9]. For decades, maxillary dentition has been widely used as a classic biomechanical research model [10–14]. In 2009, Kravitz et al. [6] conducted a prospective clinical study to address the issue of the efficacy of clear aligners. They showed that molar extrusion and mandibular incisor intrusion with clear aligners are difficult to control Thus, clear aligners have low efficiency in deep overbite correctionTo date, mandibular models have rarely been evaluated, especially the models with deep curve of Spee.

Nonsurgical deep bite correction techniques include molar extrusion, incisor intrusion, or a combination of both [15]. Extrusion of posterior teeth might be viewed as a good option in low-angle growing patients. With clear aligners, the mandibular incisors can be intruded earlier in the treatment process, allowing for more efficient and timely correction of the deep bite [16].

Extrusion is the least accurate tooth movement achieved with clear aligners. Following the distalization of the mandibular molars, there is slight labial tipping, intrusion, and protrusion of the mandibular incisors [9, 17]. Traditional orthodontic treatments often require aligning and levelling the dental arch to level the curve of Spee before placing reverse-curve arch wires [18]. Many studies have reported that movement of mandibular anterior intrusion and molar extrusion has low efficiency [6, 19, 20], especially the hypodivergent face.

Here we aimed to explore the effects of different movement patterns of mandibular molars using a three-dimensional (3D) finite element model of invisible aligners without brackets on the anterior teeth and to provide a reference for the selection of clinical treatment methods.

## Materials and methods

### Modelling reconstruction from CBCT

The 3D geometrical model was constructed using cone-beam computerized tomography (CBCT) data from a patient with a deep overbite. The study protocol was approved by the Ethics Committee of the DaLian Stomatological Hospital (DLKQLL202302). The subject provided written informed consent. All methods were performed in accordance with the relevant guidelines and regulations.

The authors confirmed that the patient provided informed consent for data collection. The CBCT images (slice thickness 0.2 mm and pixel size 0.200 mm) were input into Mimics 20.0 software (Materialise NV, Belgium) as digital imaging and communications in medicine (DICOM) files for 3D model reconstruction. For further noise reduction and smoothing, a standard tessellation language (STL) file was input into Geomagic Studio 2014 software (3D System, USA) and NX 1911 software (Siemens, Germany).

### Finite element modelling

**Fig. 1:**
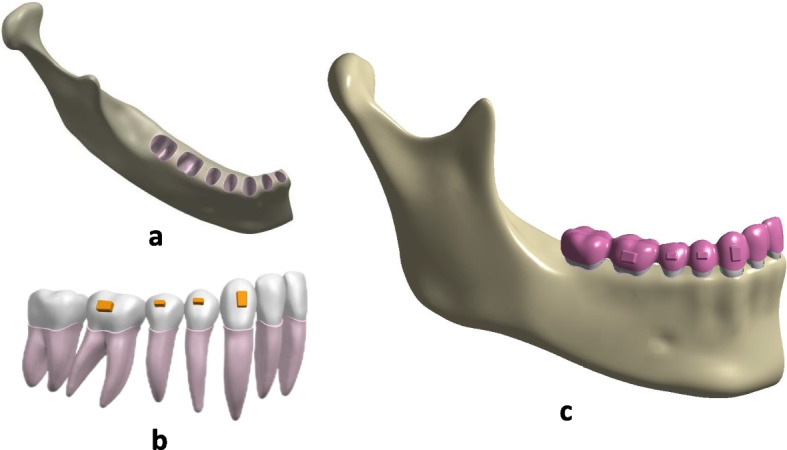
Finite models. **a** Mandibular. **b** periodontal ligament, dentition, and attachments. **c** Model of the mandibular, periodontal ligament, dentition, attachments and clear aligner

Specialized FE software, ANSYS Workbench 2019 (ANSYS, USA), was used to create the models. In the models, isotropic, homogeneous, continuous elastic materials were defined and used; only the right side of the mandibular dentition was modelled because of the models’ bilateral symmetry (Fig. 1).

To achieve greater simulation accuracy, the interface between the aligner with the tooth crown surface and the attachments was set to be in frictional contact with a friction coefficient of 0.2 [21, 22]. Additionally, friction with a coefficient of 0.18 was established at the contact interfaces between the tooth crown surface [22].

The junctions between other modelled components, such as periodontal ligament (PDL)-bone and tooth-PDL, were set to be rigidly connected. The PDL was modelled as a linear elastic material of 0.25 mm thickness [11]. The Young’s modulus and Poisson’s ratio were 0.69 MPa and 0.49, respectively. As shown in Table 1, the material properties of the components were set as those reported in previous studies [23–25].

**Table 1:** Material properties

Component	Young’s Modulus (MPa)	Poisson’s Ratio
Cortical bone	13,700	0.26
Spongy bone	1370	0.3
Teeth	19,600	0.3
Periodontium	0.69	0.49
Aligner	528	0.36
Attachment	12,500	0.36

The aligner was developed by making an external offset with a thickness of 0.5 mm according to the result of repeated measurements [23].

The numbers of nodes and elements for all the components are summarized in Table 2.

**Table 2:** Number of nodes and elements of the components of the finite element model

Component	Elements	Nodes
Cortical bone	21,036	41,723
Spongy bone	30,378	50,058
Aligner	17,659	34,514
Bone	51,414	80,515

### Establishment of a coordinate system

**Fig. 2:**
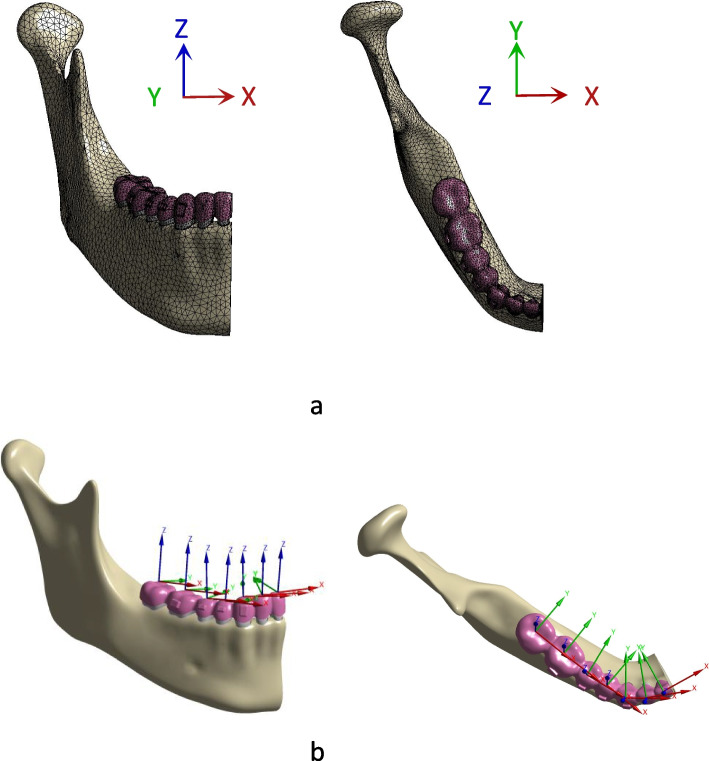
Coordinate systems. **a** Global coordinates. **b** Local coordinates. The x-, y-, and z-axes represent the labial lingual, mesiodistal, and tooth long-axis directions, respectively

This research involved the utilization of two coordinate systems (Fig. 2). First, the global coordinate system was established from CBCT data.

In the global coordinate system, the X-axis represents the direction of the coronal plane, with the positive direction pointing towards the mesial surface of the tooth; the Y-axis represents the sagittal plane, with the positive direction pointing towards the lingual surface; and the Z-axis represents the vertical plane, with the positive direction being towards the tooth crown surface.

Local coordinates for every tooth were also defined for calculating 3D movements. The local coordinates were separately located at the body centre of the clinical crown. The x-, y-, and z-axes represent the labial lingual, mesiodistal, and tooth long-axis directions, respectively.

### Experimental design

In this study, our aim was to investigate the most effective method of invisible orthodontic treatment by extruding the posterior teeth and subsequently distalizing the second molar. The configuration parameters of the five movement protocols are summarized in Table 3.

**Table 3:** Five movement protocols

	Configuration A	Configuration B	Configuration C	Configuration D	Configuration E
Distalization (0.25 mm)	7	7	7	7	7
Extrusion (0.15 mm)	–	6	4,5	4,5,6	4,5,6
Expansion (0.15 mm)	–	–	–	–	4,5,6

7=second molar; 6=first molar; 5=second premolar; 4=first premolar

## Results

**Fig. 3:**
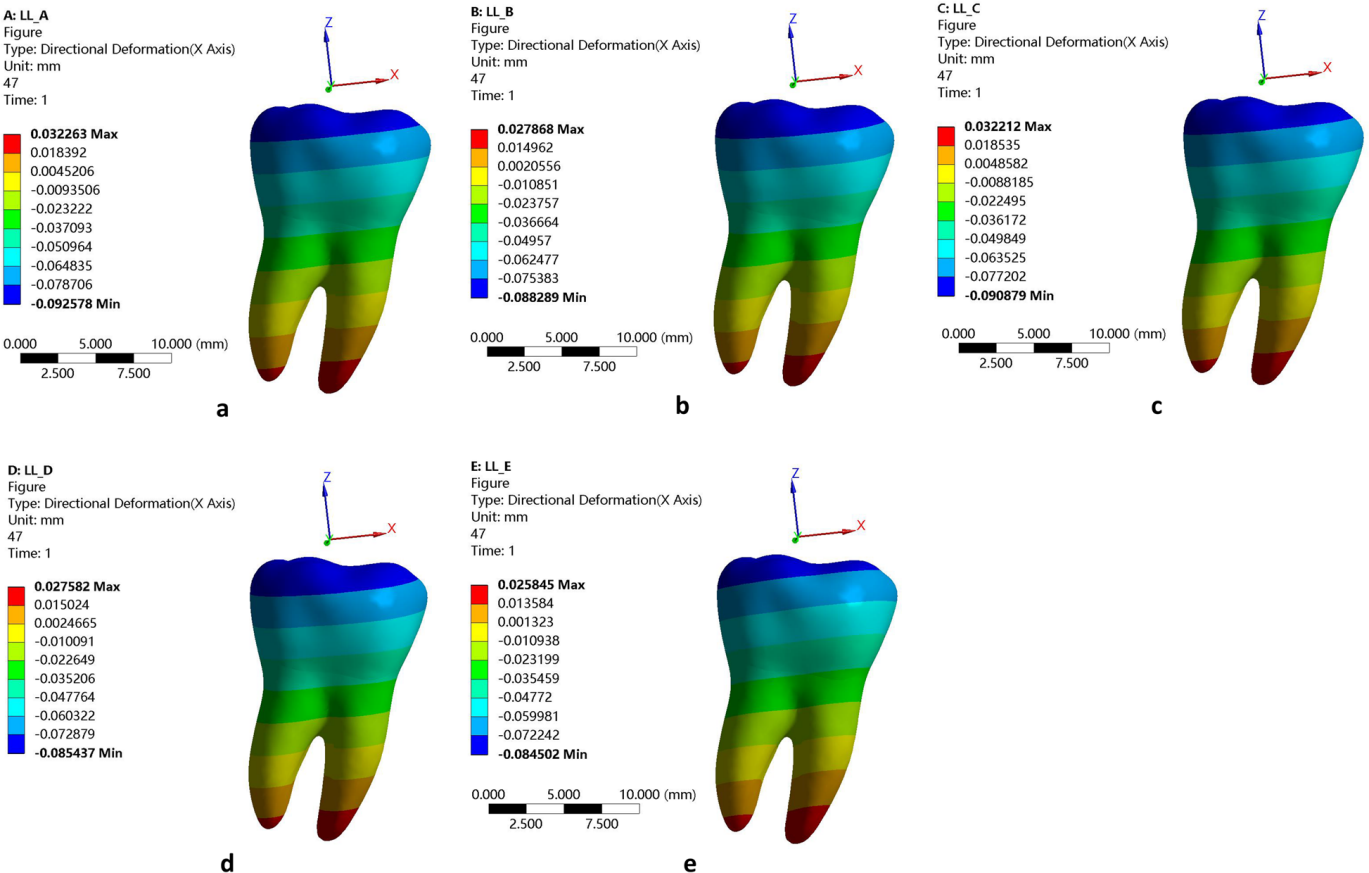
Displacement pattern of the second molar in each direction for the 5 molar displacement protocols. **a** Configuration A, (**b**) Configuration B, (**c**) Configuration C, (**d**) Configuration D and (**e**) Configuration E. The color intensity correlates with the magnitude of tooth movement, with cooler hues indicating smaller movement trends and warmer hues indicating greater movement trends. Positive and negative values denote specific directions

The results of the displacement pattern of the 2nd molar in each direction for the 5 molar displacement protocols are shown in Fig. 3.

In Configuration A, the maximum deformation observed was 0.092578 mm, followed by configurations C (0.090879 mm), B (0.088289 mm), D (0.085437 mm), and E (0.315 mm). All the root apexes moved in the mesial direction, resulting in uncontrolled lingual distal tipping movement (Fig. 3).

**Fig. 4:**
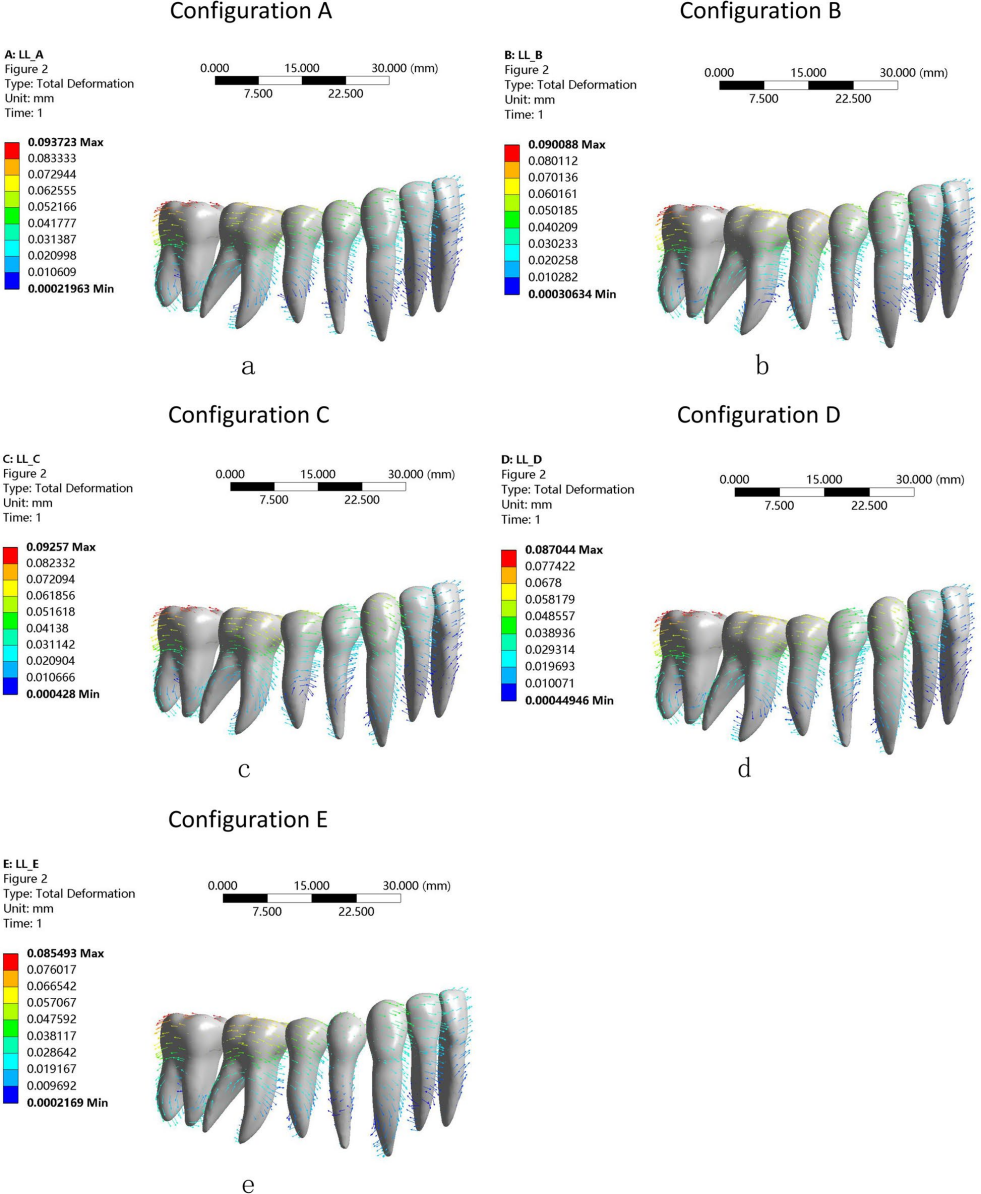
Trends in total displacement (mm) of the dentition for each configuration. Arrows indicate the direction of tooth movement; (**a**) Configuration A, (**b**) Configuration B, (**c**) Configuration C, (**d**) Configuration D and (**e**) Configuration E. the color intensity reflects the magnitude of tooth movement, with cooler hues indicating smaller movement trends and warmer hues indicating greater movement trends

Figure 4 shows the vector analysis of the overall displacement pattern of the dentition for the 5 molar distalization protocols.

The direction of movement for the second molar was mainly along the x-axis (Fig. 4). The maximum tooth displacement was obtained with the distalization configuration (configuration A), which showed 0.093723 mm of translation compared to 0.085493 mm obtained with the expansion configuration (configuration E). The second molar showed a distal inclination tendency. The mandibular anterior teeth exhibited mesial and labial proclination with a rotation centre at the intersection of the apical and middle thirds of the roots in all of the models. Uncontrolled tipping movement was observed for the entire dentition (Fig. 4).

**Fig. 5:**
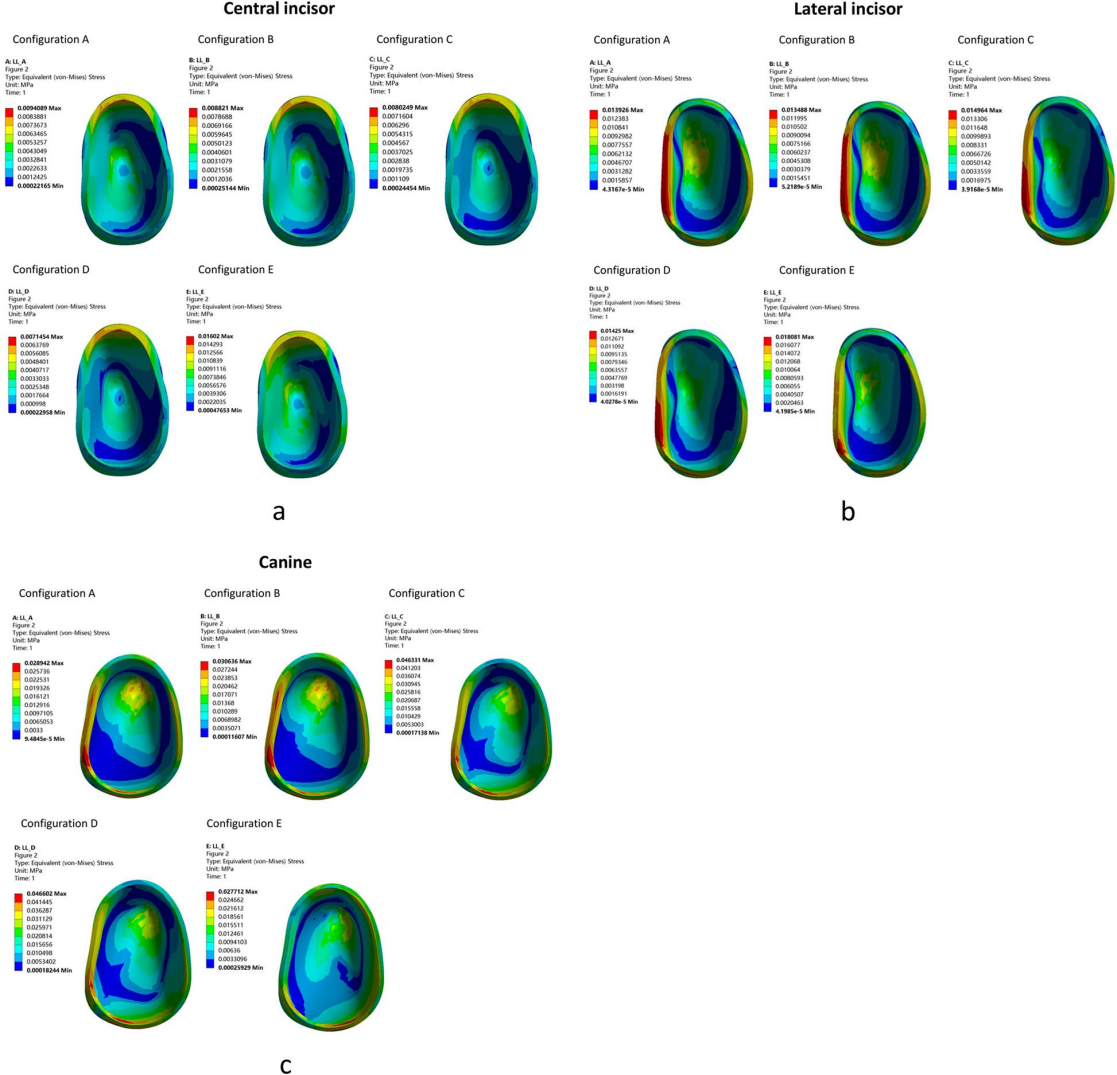
Equivalent (von Mises) stress distribution of the PDL of the anterior teeth. **a** Central incisor, (**b**) Lateral incisor, and (**c**) Canine

**Fig. 6:**
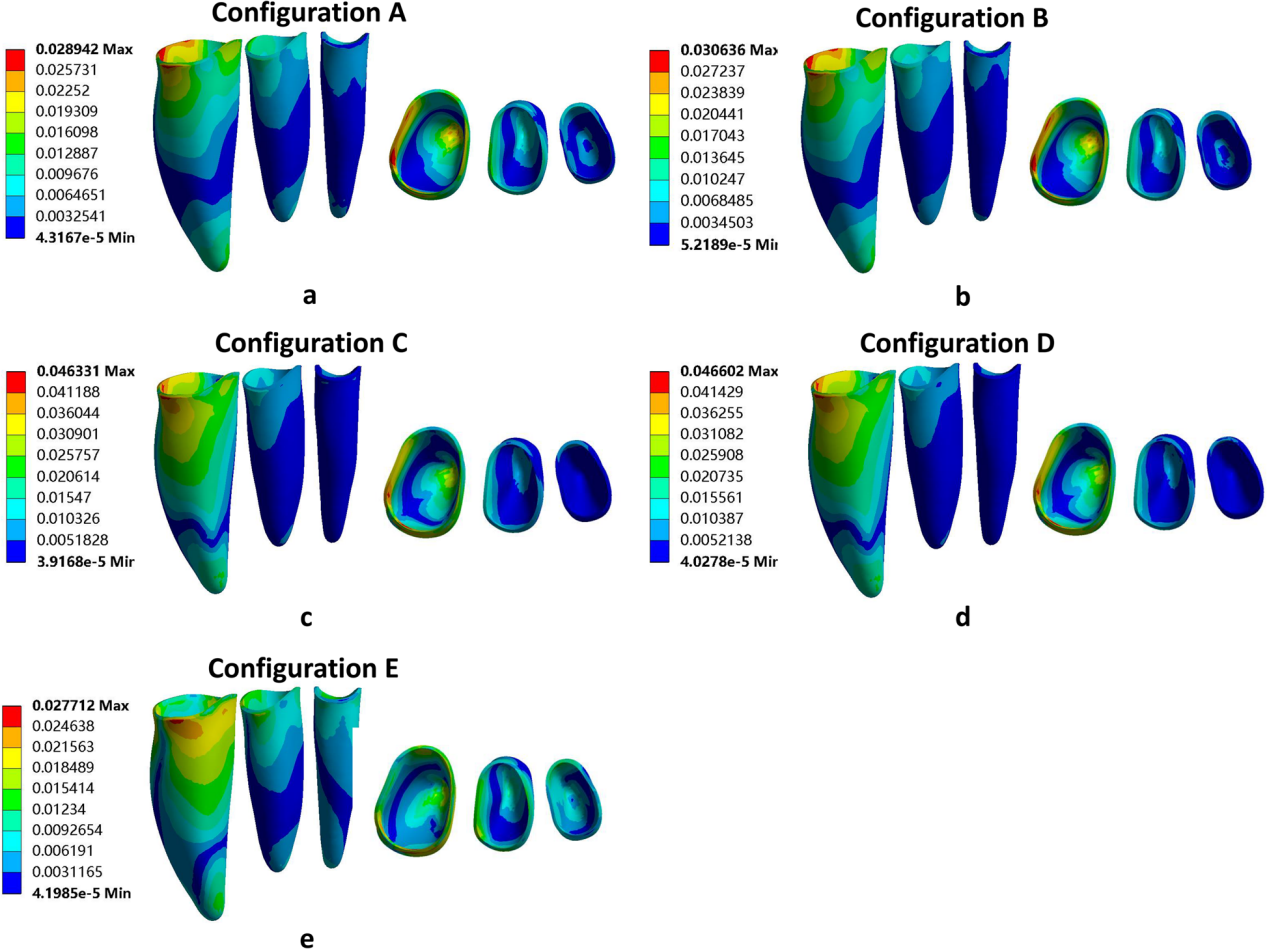
Equivalent stress distribution of the PDL of the anterior teeth (1-3). **a** Configuration A, (**b**) Configuration B, (**c**) Configuration C, (**d**) Configuration D and (**e**) Configuration E

Figures 5 and 6 show the equivalent (von Mises) stress distribution of the PDL of the central incisor, lateral incisor, and canine.

For central incisors, the stress was distributed at the cervical third on the lingual surface. In the case of lateral incisors, the stress was distributed at the cervical area on the labial and distal surfaces. However, the stress distribution for canines varied across the five groups (Fig. 5).

In configurations A and B, the stress was distributed at the cervical third and apex. In configurations C and D, the stress was distributed at the cervical area on the labial and distal surfaces, respectively, and at the apex. In configuration E, the stress was distributed in the cervical area on the labial and lingual surfaces (Fig. 5).

In the dentition of anterior teeth, the equivalent stress on the PDL of configurations A, B, C, and D was located at the distal and labial cervical area of the canine, and to a lesser extent, the root apex of the canine. In configuration E, the equivalent stress on the PDL was located at the labial cervical area of the canine (Fig. 6).

**Fig. 7:**
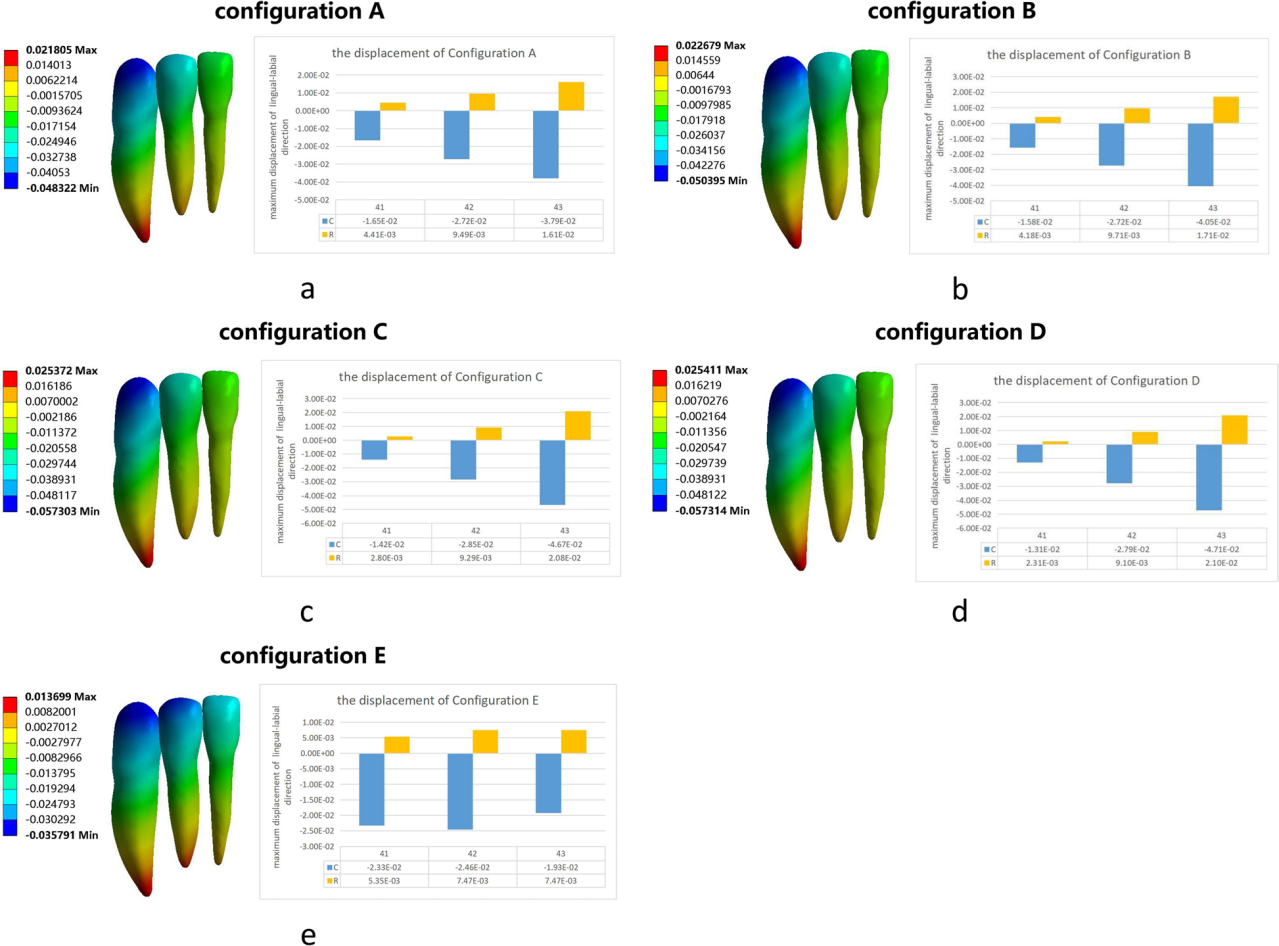
Anterior displacement in the lingual-labial direction. **a** Configuration A, (**b**) Configuration B, (**c**) Configuration C, (**d**) Configuration D and (**e**) Configuration E

The maximum labial displacement of the central incisor crown was observed in configuration E, and the maximum labial displacement of the lateral incisor and canine was found in configurations C and D, respectively (Fig. 7).

## Discussion

Deep bite malocclusions are a relatively common type of malocclusion found in adolescents and adults, and their severity can be influenced by a combination of dental and skeletal conditions [26]. Skeletal deep bite malocclusion is predominantly characterized by a horizontal growth pattern of the mandible, resulting in a lower anterior face height and a deep curve of Spee [27, 28]. In these cases, extrusion of posterior tooth resulted in an increase in the posterior vertical dimension. On the other hand, dental deep bite malocclusions are caused by excessive development of the alveolar bone in the anterior teeth region or insufficient development of the alveolar bone in the posterior teeth region. Somes studies have indicated that both maxillary and mandibular anterior intrusion were inaccurate [20, 29], Shahabuddin found approximately 1 mm of intrusion of mandibular incisors with 42.5% accuracy [20]; in other words,correcting deep overbite and deep curve of Spee by only intruding the mandibular anterior teeth is not an efficient method, The main mechanisms of deep bite correction were proclination of incisors, intrusion of incisors, and extrusion of mandibular posterior teeth. Depending on whether deep bite malocclusions have a skeletal or dental aetiology, corrective techniques that involve maxillary and mandibular incisor intrusion, proclination, posterior tooth extrusion, and increasing lower anterior facial height through surgical means have been proven effective [15, 30]. It may increase efficacy to intrude the incisor and extrude the posterior at the same time while treating skeletal class II with a low-angle of the deep curve of Spee.

Correcting the deep curve of Spee via extrusion of mandibular posterior teeth has poor efficiency, but Khosravi et al. [31] reported that an approximately 1.5 mm overbite improvement could be achieved when Invisalign appliances were used by patients with a deep overbite. Additionally, clear aligners could improve vertical dimensional malocclusions, such as dental deep bites, within a mild to moderate range.

Our results for the displacement pattern of the second molar demonstrate that extruding the premolars during molar distalization has little impact on molar distalization (Fig. 3). According to the initial displacement results shown in Fig. 3, the greatest maximum tooth displacement (0.092578 mm) was obtained by configuration A (distalization of the second molar), while the lowest (0.084502 mm) was obtained by configuration E (distalization, extrusion and expansion), representing a 0.008076 mm difference. The tooth displacement of configuration C (extrusion of 4 and 5 simultaneously) was 0.090879 mm, differing from that of configuration A by 0.001699 mm. However, it’s important to note that the maximum tooth displacement alone does not provide comprehensive information for analyzing the efficacy of tooth movement as it does not offer insights into the movement direction.

As shown in Fig. 3, it was observed that the second molar in the mesial-distal (x-axis) direction underwent the maximum distal displacement at every point in the distalization protocol (configuration A). Each configuration exhibited a certain degree of rotation in the second molars during distalization. The results shown in Figs. 3 and 4 are essentially consistent. The difference between configuration A (distalization group) and configuration C (extrusion of 4 and 5 simultaneously) was not significant, indicating that the simultaneous distalization of the second molar and extrusion of premolars has a relatively minor impact on distalization. With the extrusion of the posterior teeth, the expansion of the arch had little effect on distalization (Figs. 3 and 4). Therefore, in clinical practice, it is possible to simultaneously extrude the premolar teeth while distalizing the molars to improve the efficiency of tooth movement.

The anchorage loss of the anterior tooth in orthodontic treatment can lead to labial inclination of the incisors, thereby affecting the efficiency of molar distalization [32, 33]. According to the principle of force interaction,clear aligners are subjected to opposite forces on the anterior teeth during molar distalization [12, 14]. To increase anterior tooth anchorage, clear aligners perform sequential molar distalization, utilizing the remaining teeth as a group anchor to counteract the reactive forces generated during the distalization process [34]. Furthermore studies [35, 36] suggest that elastic traction and mini-implants can be beneficial in enhancing anterior tooth anchorage control during molar distalization with clear aligners. Our studies indicated that distalization and expansion may not increase anchorage loss of anterior teeth (Figs. 4, 7). In patients who require levelling of the Spee curve, when sequencing the distalization of the second molar, the teeth anterior to the first molar could maintain immobility in the sagittal direction and can be used as an anchor to enhance the clinical efficacy of tooth movement. The premolars and the first molar can be simultaneously extruded in the vertical direction to improve efficiency.

Alveolar bone resorption, a potential side effect of orthodontic treatment, arises from excessive concentrated stress [37, 38]. After orthodontic treatment, patients with significant alveolar ridge resorption may have a higher incidence of gingival recession [39, 40]. Some studies have demonstrated a higher incidence of gingival recession with labial-tipping movements [41].

Based on our experiments, clear aligners produced high levels of concentrated stress mainly on the cervical area, indicating that the buccal alveoli fossa might bear higher stress and is prone to the risk of buccal alveoli fossa resorption and gingival recession (Fig. 5). In our study, the equivalent stress on the periodontal membrane of the canines in configurations C and D was twice that of configuration A (Figs. 5 and 6). However, the equivalent stress distribution in the PDL of configurations C and D was more scattered than that of the other configurations (Fig. 6). In configuration E (distalization, extrusion and expansion), the central incisors were subjected to significantly greater stress than in the other configurations, and the lateral incisors were also found to be stressed to a greater extent. Comparing the results for configurations D and E, there is a greater force on the incisors during distal movement and expansion of the arch (Fig. 6). For patients with a thin alveolar bone in their anterior teeth, it is important to prevent buccal bone resorption and gingival recession during the process. In configuration E, there was a concentration of stress on the buccal cervical area of the canines (Fig. 6). During expansion, it is necessary to monitor the condition of the buccal alveolar bone. In the anterior dentition, the maximum stress was concentrated on the cervical area of the canines in all configurations. This indicates that during orthodontic treatment, attention should be given to the cervical area of the canines. As a result, the anchorage loss of anterior teeth in orthodontics can lead to increased labial tipping and protrusion of the maxillary incisors [32, 33], which can affect the efficiency of molar distalization. Whether the patient has good periodontal health or is suffering from periodontal disease, it is important to protect the canines when retracting the upper anterior teeth, ultimately avoiding excessive forces on the canines that can lead to gingival recession, alveolar bone resorption, dehiscence, and fenestration.

This study has several limitations. First,it is limited by the current finite element analysis technology. Tooth movement is influenced by many factors,especially the condition of periodontal tissue. The health of the periodontal tissue directly impacts the design method and magnitude of molar movement. When the lower anterior alveolar bone is reduced, distalization of mandibular molars may affect the machanism of lower anterior teeth. Second, this study includes a single model rather than a sample of models with varied severities. During the process of distalization of the mandibular molars, different curves of Spee may have different modalities. In the future, more animal and clinical experiments should be carried out to acquire evidence at a higher level.

## Conclusions

In conclusion, this study showed the following: (1) All different patterns forcorrecting the curve of Spee with clear aligners results anchorage loss of the anterior teeth. During this process, the anterior teeth exhibited labial inclination with a rotation centre at the intersection of the apical and middle thirds of the roots. (2) Simultaneously extruding the premolars during molar distalization has little impact on molar distalization (3) When expansion and extrusion occur simultaneously during the distalization of teeth, attention must be given to the alveolar bone on the labial side of anterior teeth, especially the canines, to prevent the occurrence of gingival recession, dehiscence, and fenestration.

## Data Availability

The datasets used and/or analysed during the current study are available from. The corresponding author on reasonable request.

## Abbreviations

FE: Finite element
FEA: Finite element analysis
PDL: Periodontal ligament
CBCT: Cone beam computed tomography

## References

[1] Johal A, Bondemark L (2021). Clear aligner orthodontic treatment: angle Society of Europe consensus viewpoint. J Orthod.

[2] Gu J, Tang JS, Skulski B, Fields HW, Beck FM, Firestone AR, Kim DG, Deguchi T (2017). Evaluation of Invisalign treatment effectiveness and efficiency compared with conventional fixed appliances using the peer assessment rating index. Am J Orthod Dentofac Orthop.

[3] Gao M, Yan X, Zhao R, Shan Y, Chen Y, Jian F, Long H, Lai W (2021). Comparison of pain perception, anxiety, and impacts on oral health-related quality of life between patients receiving clear aligners and fixed appliances during the initial stage of orthodontic treatment. Eur J Orthod.

[4] Ben Gassem AA (2022). Does clear aligner treatment result in different patient perceptions of treatment process and outcomes compared to conventional/traditional fixed appliance treatment: a literature review. Eur J Dent.

[5] Bichu YM, Alwafi A, Liu X, Andrews J, Ludwig B, Bichu AY, Zou B (2023). Advances in orthodontic clear aligner materials. Bioact Mater.

[6] Kravitz ND, Kusnoto B, BeGole E, Obrez A, Agran B (2009). How well does Invisalign work? A prospective clinical study evaluating the efficacy of tooth movement with Invisalign. Am J Orthod Dentofac Orthop.

[7] Haouili N, Kravitz ND, Vaid NR, Ferguson DJ, Makki L (2020). Has Invisalign improved? A prospective follow-up study on the efficacy of tooth movement with Invisalign. Am J Orthod Dentofac Orthop.

[8] Simon M, Keilig L, Schwarze J, Jung BA, Bourauel C (2014). Treatment outcome and efficacy of an aligner technique--regarding incisor torque, premolar derotation and molar distalization. BMC Oral Health.

[9] Wu D, Zhao Y, Ma M, Zhang Q, Lei H, Wang Y, Li Y, Chen X (2021). Efficacy of mandibular molar distalization by clear aligner treatment. Zhong Nan Da Xue Xue Bao Yi Xue Ban.

[10] Kwak KH, Oh S, Choi YK, Kim SH, Kim SS, Park SB, Kim YI (2023). Effects of different distalization directions and methods on maxillary total distalization with clear aligners: a finite element study. Angle Orthod.

[11] Jiang T, Wu RY, Wang JK, Wang HH, Tang GH (2020). Clear aligners for maxillary anterior en masse retraction: a 3D finite element study. Sci Rep.

[12] Fontana M, Cozzani M, Caprioglio A (2012). Non-compliance maxillary molar distalizing appliances: an overview of the last decade. Prog Orthod.

[13] Kawamura J, Park JH, Kojima Y, Tamaya N, Kook YA, Kyung HM, Chae JM (2021). Biomechanical analysis for total distalization of the maxillary dentition: a finite element study. Am J Orthod Dentofac Orthop.

[14] Fuziy A, Rodrigues de Almeida R, Janson G, Angelieri F, Pinzan A (2006). Sagittal, vertical, and transverse changes consequent to maxillary molar distalization with the pendulum appliance. Am J Orthod Dentofac Orthop.

[15] Parker CD, Nanda RS, Currier GF (1995). Skeletal and dental changes associated with the treatment of deep bite malocclusion. Am J Orthod Dentofac Orthop.

[16] Henick D, Dayan W, Dunford R, Warunek S, Al-Jewair T (2021). Effects of Invisalign (G5) with virtual bite ramps for skeletal deep overbite malocclusion correction in adults. Angle Orthod.

[17] Bowman SJ (2017). Improving the predictability of clear aligners. Semin Orthod.

[18] Rozzi M, Tiberti G, Mucedero M, Cozza P (2022). Leveling the curve of Spee: comparison between continuous archwire treatment and Invisalign system: a retrospective study. Am J Orthod Dentofac Orthop.

[19] Krieger E, Seiferth J, Saric I, Jung BA, Wehrbein H (2011). Accuracy of Invisalign® treatments in the anterior tooth region. First results. J Orofac Orthop.

[20] Shahabuddin N, Kang J, Jeon HH (2023). Predictability of the deep overbite correction using clear aligners. Am J Orthod Dentofac Orthop.

[21] Ramalho A, Antunes PV (2007). Reciprocating wear test of dental composites against human teeth and glass. Wear.

[22] Shao C, Jin B, Mu Z, Lu H, Zhao Y, Wu Z, Yan L, Zhang Z, Zhou Y, Pan H, et al. Repair of tooth enamel by a biomimetic mineralization frontier ensuring epitaxial growth. Sci Adv. 2019;5(8):eaaw9569. 10.1126/sciadv.aaw9569 PMC6716959 31497647

[23] Cortona A, Rossini G, Parrini S, Deregibus A, Castroflorio T (2020). Clear aligner orthodontic therapy of rotated mandibular round-shaped teeth: a finite element study. Angle Orthod.

[24] Gomez JP, Pena FM, Martinez V, Giraldo DC, Cardona CI (2015). Initial force systems during bodily tooth movement with plastic aligners and composite attachments: a three-dimensional finite element analysis. Angle Orthod.

[25] Liang W, Rong Q, Lin J, Xu B (2009). Torque control of the maxillary incisors in lingual and labial orthodontics: a 3-dimensional finite element analysis. Am J Orthod Dentofac Orthop.

[26] Danz JC, Greuter C, Sifakakis I, Fayed M, Pandis N, Katsaros C (2014). Stability and relapse after orthodontic treatment of deep bite cases-a long-term follow-up study. Eur J Orthod.

[27] Bjork A (1955). Facial growth in man, studied with the aid of metallic implants. Acta Odontol Scand.

[28] Björk A (1969). Prediction of mandibular growth rotation. Am J Orthod.

[29] Al-Balaa M, Li H, Ma Mohamed A, Xia L, Liu W, Chen Y, Omran T, Li S, Hua X (2021). Predicted and actual outcome of anterior intrusion with Invisalign assessed with cone-beam computed tomography. Am J Orthod Dentofac Orthop.

[30] Nanda R (1997). Correction of deep overbite in adults. Dent Clin N Am.

[31] Khosravi R, Cohanim B, Hujoel P, Daher S, Neal M, Liu W, Huang G (2017). Management of overbite with the Invisalign appliance. Am J Orthod Dentofac Orthop.

[32] Hilgers JJ (1992). The pendulum appliance for class II non-compliance therapy. J Clin Orthod.

[33] Bussick TJ, McNamara JA (2000). Dentoalveolar and skeletal changes associated with the pendulum appliance. Am J Orthod Dentofac Orthop.

[34] Lai WL (2017). Extraction cases using clear aligners. Zhonghua Kou Qiang Yi Xue Za Zhi.

[35] Bowman SJ, Celenza F, Sparaga J, Papadopoulos MA, Ojima K, Lin JC (2015). Creative adjuncts for clear aligners, part 1: class II treatment. J Clin Orthod.

[36] Liu LXGY, Wu XP (2021). Research progress of molar distalization with clear aligner orthodontics. Stomatology..

[37] Katsaros IJ-VCGNTASC (2010). Orthodontic therapy and gingival recession:a systematic review. Orthod Craniofac Res.

[38] Kassab MM, Cohen RE. The etiology and prevalence of gingival recession. J Am Dent Assoc. 2003;134(2):220–5. 10.14219/jada.archive.2003.0137 12636127

[39] Tarnow DP, Magner AW, Fletcher P (1992). The effect of the distance from the contact point to the crest of bone on the presence or absence of the interproximal dental papilla. J Periodontol.

[40] Liu Lin KI, Mitani H (2007). Hanges of anterior papilla after orthodontic treatment. Chin J Orthod..

[41] Kandasamy S, Goonewardene M, Tennant M (2007). Changes in interdental papillae heights following alignment of anterior teeth. Aust Orthod J.

